# Whole Body Melanoma Transcriptome Response in Medaka

**DOI:** 10.1371/journal.pone.0143057

**Published:** 2015-12-29

**Authors:** Manfred Schartl, Yingjia Shen, Katja Maurus, Ron Walter, Chad Tomlinson, Richard K. Wilson, John Postlethwait, Wesley C. Warren

**Affiliations:** 1 Physiological Chemistry, University of Würzburg, Biozentrum, Am Hubland, 97074, Würzburg, Germany; 2 Comprehensive Cancer Center, University Clinic Würzburg, Josef Schneider Straße 6, 97074, Würzburg, Germany; 3 Department of Chemistry and Biochemistry, 419 Centennial Hall, Texas State University, 601 University Drive, San Marcos, TX, 78666, United States of America; 4 McDonnell Genome Institute at Washington University, 4444 Forest Park Blvd., St Louis, MO, 63108, United States of America; 5 Institute of Neuroscience, University of Oregon, 1425 E. 13th Avenue, Eugene, OR, 97403, United States of America; University of Alabama at Birmingham, UNITED STATES

## Abstract

The incidence of malignant melanoma continues to increase each year with poor prognosis for survival in many relapse cases. To reverse this trend, whole body response measures are needed to discover collaborative paths to primary and secondary malignancy. Several species of fish provide excellent melanoma models because fish and human melanocytes both appear in the epidermis, and fish and human pigment cell tumors share conserved gene expression signatures. For the first time, we have examined the whole body transcriptome response to invasive melanoma as a prelude to using transcriptome profiling to screen for drugs in a medaka (*Oryzias latipes*) model. We generated RNA-seq data from whole body RNA isolates for controls and melanoma fish. After testing for differential expression, 396 genes had significantly different expression (adjusted p-value <0.02) in the whole body transcriptome between melanoma and control fish; 379 of these genes were matched to human orthologs with 233 having annotated human gene symbols and 14 matched genes that contain putative deleterious variants in human melanoma at varying levels of recurrence. A detailed canonical pathway evaluation for significant enrichment showed the top scoring pathway to be antigen presentation but also included the expected melanocyte development and pigmentation signaling pathway. Results revealed a profound down-regulation of genes involved in the immune response, especially the innate immune system. We hypothesize that the developing melanoma actively suppresses the immune system responses of the body in reacting to the invasive malignancy, and that this mal-adaptive response contributes to disease progression, a result that suggests our whole-body transcriptomic approach merits further use. In these findings, we also observed novel genes not yet identified in human melanoma expression studies and uncovered known and new candidate drug targets for further testing in this malignant melanoma medaka model.

## Introduction

While the incidence of most cancers is decreasing, malignant melanoma (MM) continues to increase by 3–7% per year in Caucasians and has a 5-year survival rate of only 20% [1]. Melanoma genome sequencing and exome sequencing of an astonishingly high heterogeneity of clinical samples have led to the identification of a large catalog of recurrent somatic variants [2], most notably *BRAF* and *NRAS*. However, patient outcomes from treatment with novel compounds are compromised by an almost 100% recurrence rate due to rapidly occurring drug resistance [3] and melanoma suppression remains unpredictable when targeting protein-coding genes identified in large cohort sequencing projects. A deeper exploration of cause-and-effect melanomagenesis data is urgently needed. At present, vertebrate melanoma models exist for zebrafish, medaka, platyfish, mouse, the Syrian hamster, Mongolian gerbil and opossum [4–7], each with its own advantages and disadvantages for utility in drug discovery and molecular mimicry of human melanoma.

In contrast to humans, mice and other rodent skin is devoid of epidermal melanocytes as they occupy the hair follicles and dermis, except for some in the skin of footpads, tail, ears and snout, whereas fish melanoma models mimic the appearance of melanocytes in the human epidermis. Another advantage appears to be the lack of hair cycling which periodically changes the profile of gene expression in skin and other organs, which complicates studies on tumor environment. Fish models available in zebrafish and medaka have additional advantages, including easy transgenesis, superior suitability for bioimaging analyses due to the transparency of embryos and larvae and importantly, their suitability for high throughput chemical screens [8, 9]. While single driver gene melanoma models, such as medaka and zebrafish, are initially genetically less complex than human melanomas, the expression signatures of zebrafish and medaka melanomas clearly show common features with human melanoma [10, 11]. Fish models have proven their value for melanoma research and have become well-accepted tools for furthering our knowledge and for developing better diagnostic and therapeutic tools [7, 12].

Several studies have examined gene expression changes in zebrafish, medaka, mouse and human melanomas [13–15]. A recent meta-analysis of human melanoma microarray data suggested that 17 genes show aberrant expression [15]. All approaches [13–15] thus far reported have dissected out melanoma tumor cells of various classifications, mostly from the metastatic disease, and compared results to normal skin or melanocytic nevi. None of these studies measured the whole-body transcriptome in established melanoma and compared results to normal individuals. This type of comparison is necessary because tumors also have effects on entire organisms; furthermore, tissue systems throughout the organism play a role in tumor growth and disease, especially the vasculature, endocrine, and immune systems [12, 16]. Melanoma is a highly invasive and metastatic tumor where already early on, small nests or single tumor cells colonize the whole body. This is further complicated by the fact that these microtumors are often non-pigmented and reside far away from the primary lesion, making them easy to overlook even by an experienced experimenter. Therefore, our strategy was to compare the transcriptomes of intact melanoma fish rather than dissecting the melanoma from the “healthy” part of the individual. This strategy allowed for the establishment of a melanoma transcriptional disease signature and identified disregulation of various canonical pathways that may indicate a systemic response to the spread of malignant melanocytes.

## Material and Methods

### Sequencing

A transgenic melanoma model in medaka (*Oryzias latipes*) was used to determine the transcriptional disease signature (TDS) of melanoma. In this model, the oncogenic receptor tyrosine kinase Xmrk, which is encoded by the melanoma-inducing gene from the platyfish (*Xiphophorus maculatus*), is specifically expressed in pigment cells under control of the medaka *mitf* promoter [17]. The stably integrated transgene was introduced into an inbred strain (Carbio) of medaka by introgressive breeding. Fish were kept under standard conditions in the aquarium facility of the Biozentrum at the University of Würzburg and all studies were approved by the Institutional Review Board. For RNA-seq experimentation, total RNA was extracted from the intact bodies of size-matched (1cm total length) non-transgenic and transgenic fish 3–5 weeks old using an RNeasy Qiagen kit (Venlo, Netherlands) according to the manufacturers’ protocol. To compare gene expression between controls and melanoma fish, a biological sample was prepared and sequenced individually from four individual melanoma fish and four individual control fish, thus providing four independent biological replicates of each condition. We checked total RNA for quality on the Agilent Fragment Analyzer, then enriched for poly(A)+ RNA using the MicroPolyA Purist kit (Ambion, Carlsbad, CA). We used ScripSeq (Epicentre, Madison, WI) to generate strand-specific cDNA that was sequenced on the Illumina Hiseq2000 platform as 100 base paired-end reads (insert size of 400bp). All sequences are available under accession number PRJNA219474.

### Differential gene expression

Across samples, an average of 34.5 million read pairs with average base quality >35.7 were obtained after filtering for duplicates and low quality bases. Sequences from each sample were mapped to medaka transcripts (Ensembl version 80) using TopHat2 [18]. A custom perl script, available on request, was used to count mapped reads per each transcript in each sample. DESeq2 was used to analyze read count data from the previous step and to identify differential expression levels based on an error model that uses the negative binomial distribution[19]. In this study for all control to tumor gene comparisons we used an adjusted p-value of <0.02 as our significance threshold for further biological inference.

### RNA-seq validation

For confirmation of RNA-seq data, selected genes were assayed with qPCR from ten control and ten melanoma fish individually (siblings of fish used for the RNA-seq experiments and two melanoma fish that were also used for the RNA-seq). RNA was isolated from whole fish bodies using the RNeasy Mini Kit (Qiagen, Venlo, Netherlands) according to the manufacturers’ protocol. cDNA was prepared from total RNA using the RevertAid kit (Fermentas, Waltham, MA) with random hexamer primers. 25 ng of each cDNA was analyzed in triplicate in a 25 μl volume using a SYBR green-containing mastermix for 5 min at 95°C followed by 40 cycles of 95°C for 30 s, 60°C for 30 s and 72°C for 20 s in a mastercycler ep realplex (Eppendorf, Hamburg Germany). Expression of each gene was normalized to *ef1a1* levels, a positive control gene, and fold changes were calculated versus one wild-type fish. All p-values were calculated by applying the Mann-Whitney-U-test.

### Pathway and transcription regulator enrichment

To infer further functional information from malignant melanoma whole body transcriptomes, it was necessary to match each regulated medaka gene to its human ortholog and, when possible, affiliated human gene symbol. Of 396 differentially regulated medaka genes (adjusted p value < .02), we were able to obtain 233 medaka genes with matching human gene annotation, a prerequisite for many statistical tests of functional enrichment. We used a combination of human orthology retrieval through the Ensembl BioMart tools (a predefined set of multispecies orthologs) and manual alignment curation by BLAST to derive this set. It is recognized in some cases that an additional copy of each gene could be present in the medaka genome because teleosts experienced an additional unique whole genome duplication event [20, 21]. The human gene symbols and log ratio values for each differentially expressed medaka gene were input into the Ingenuity core analysis software [22]. We then performed canonical pathway enrichment and upstream regulatory activation analyses. To measure statistical significance for over-represented canonical pathways, we used a right-tailed Fisher’s exact test (p-value cutoff < .01). To ascertain transcription regulators that could explain observed gene expression changes, we relied on two test statistics 1) a p-value overlap score was calculated with a Fishers exact test, whereby a statistical overlap is calculated between differentially expressed genes and the known targets of the transcription regulators (p-value cutoff < .01) and 2) a activation z-score that attempts to infer activation states based on prior knowledge of regulation by the regulator compared to a random model of regulation, a z-score of greater than 2 or less than -2 were considered significant [22].

### Drug interaction

To discover genes that could be targeted with known drugs, we input the 233 medaka expression candidates with human orthologs into the DGIdb database (http://dgidb.genome.wustl.edu) and performed a search with default screen parameters to find all gene to drug interactions using the antineoplastic filter [23].

## Results

**Fig 1:**
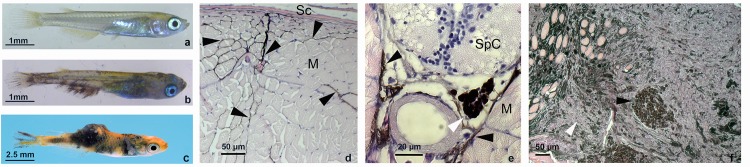
Transgenic medaka model for melanoma in juvenile fish (a) Control sibling and (b) fish with the *mitf*:*xmrk* transgene showing onset of malignant melanoma spreading over the fins and invading the body musculature at 3–4 weeks age (c) fish at 8 weeks of age with invasive and nodular melanoma. (d, e) Transverse sections through the posterior region of 4 weeks old *mitf*:*xmrk* transgenic medaka. (d) Melanoma invasion deep into the body trunk musculature. (e) Nest of melanoma cells (white arrowhead) close to the spinal cord. M muscle bundles, Sc scale, SpC spinal cord, black arrowheads point to stretches of invading melanoma cells, scale bars represent 50 μm in (d) and 20 μm in (e). (f) a nodular melanoma in an adult *mitf*:*xmrk* medaka. The white arrowhead points to an area with lowly differentiated melanoma cells and a black arrowhead indicates a nest of heavily pigmented melanoma cells.

We used our transgenic medaka model (*tg(mitf*:*xmrk))* for melanoma where the pigment cell-transforming oncogene *xmrk* [24] isolated from the platyfish *Xiphophorus maculatus* was placed under control of the medaka *mitf* (microphthalmia-associated transcription factor) promoter, which drives oncogene expression in melanocytes [17]. The *mitf* promoter confers strong but strictly pigment cell-specific expression to the tumor-inducing driver-oncogene *xmrk* with no ectopic or background expression [17, 25–27]. In the homogenous genetic background of the medaka strain used here (Fig 1A), transgenic juveniles start to develop highly invasive extracutaneous malignant melanoma at late larval stages (3–4 weeks of age) with almost 100% penetrance [17] (Fig 1B). At later stages (7–8 weeks), these melanomas progress to metastatic disease with multiple large nodular and invasive tumors in the abdomen and trunk musculature (Fig 1C). In the initial stage of melanoma development, the progression of the malignant disease is mostly apparent by invasion of tumor cells deeply into the trunk musculature, where they follow the space between single muscle bundles (Fig 1D). At multiple extracutaneous sites proliferation of melanoma cells that arrived from elsewhere leads to tumor nodules and lesion expansion (Fig 1E).

We generated RNA-seq data from whole body RNA isolates for four melanoma and four control siblings at 3–4 weeks of age, a stage when the tumors switch from localized lesions to progressive malignant disease. Transcript read counts >10 per individual in at least 50% of all fish (control and tumor) were generated for 19,985 of 25,434 Ensembl medaka genes. After read count normalization and statistical testing for differentially expressed genes [28], 396 of medaka genes were found to have significantly different expression levels (adjusted p-value<0.02) between melanoma and control fish. Among all differentially expressed genes, a total of 233 matched human orthologs with descriptive gene annotation, which allowed for putative biological inference, were considered further for statistical enrichment tests (Table A in S1 File). Independent quantitative PCR validation of a sub-set of these regulated genes confirmed the direction of expression for a majority (6 of 8; Table B in S1 File). We purposefully chose genes of importance to melanoma biology (*irf1*, *atm*), immune systems (*tlr2*, *stat4*) and others of unknown melanoma association but significantly regulated in our medaka model (*stc1*, *slc24a5*, *epd1*, *gadd45a*). The reason for non-validation is unknown, but several possibilities exist, including paralogs confounding probe hybridization kinetics.

Overall in our study, more genes were down-regulated (n = 138) than up-regulated (n = 95) in melanoma fish compared with controls (Table A in S1 File). Of this set, 14 matched human genes shown to harbor recurrent somatic mutations [29] (Table C in S1 File). The upper and lower log ratio expression extremes were +2.48 and -5.67 for *fkbp5* and *snord14c*, respectively. The role of *snord14c* in melanoma progression is unknown, as *snord14c* is a member of the small nucleolar RNAs (snoRNAs) and is classified as a C/D box snoRNA that generally are linked to methylation [30]. In contrast, *fkbp5* is a member of the immunophilin protein family, which play a role in immunoregulation and basic cellular processes involving protein folding and trafficking and has been recently linked to melanoma dissemination [31].

**Fig 2:**
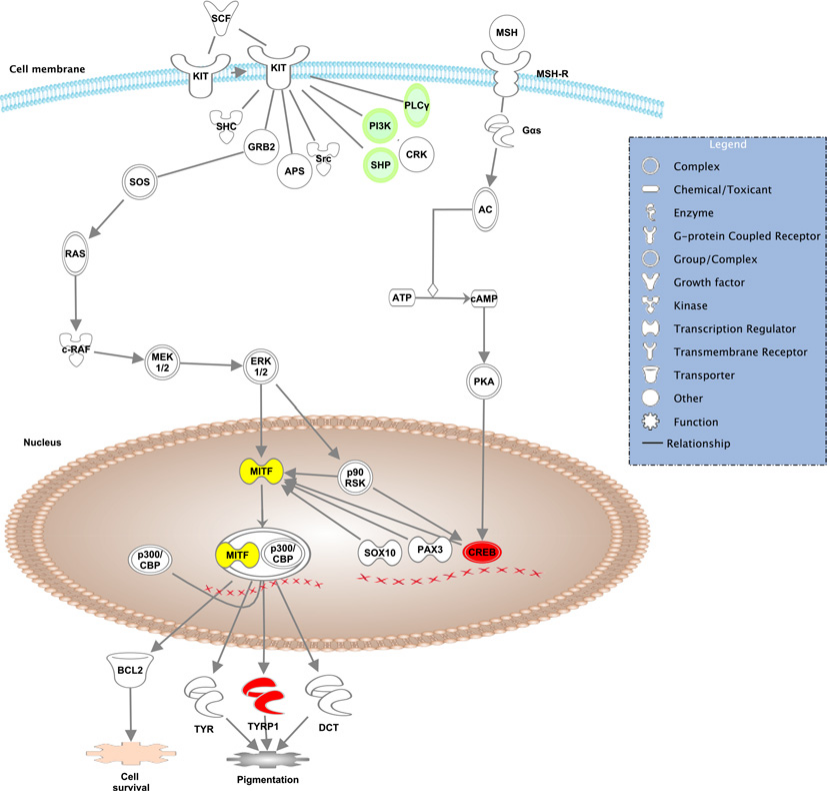
Genes known to be associated with melanocyte development and pigmentation signaling. Red and green colored gene symbols are increased or decreased, respectively, in melanoma fishes compared to control. The *mitf* gene (yellow) is upregulated but fails to reach statistical significance.

We sought to validate our whole body transcriptome model by examining expression directionality of known molecular participants of the melanocyte development and pigmentation signaling pathways. The expression levels of five gene members were significantly (adjusted p-value < .02) elevated or decreased: *shp*, *creb*, *pi3k*, *plcg2*, and *tyrp1* (Fig 2). As in other vertebrates, medaka melanomas originate from neural crest-derived pigment synthesizing cells and elevated expression of *mitf*, a key transcriptional regulator in melanoma, has been functionally linked to the neoplastic phenotype in humans[32]. However, in the whole body transcriptome, the *mitf* gene was up regulated two-fold in the melanoma fish but did not reach our p-value < .02 significance threshold (adjusted p-value 0.19). Nonetheless, the pigmentation gene *tyrp1* showed a elevated level of expression in the whole body of melanoma fish as a result of *mitf* upregulation (Fig 2; p-value <0.0001). Another upregulated gene is hypoxia inducible factor 3. In mammalian melanocytes induction of melanin pigmentation led to upregulation of *hif1*α expression and related pathways [33]. A well-established melanoma marker in dermatooncology, *pmel*, was increased as well (adjusted p-value <0.00002). A striking and novel feature not noted earlier in expression studies using dissected fish melanoma was a general down-regulation of components of the innate immune system. Some other significant examples of note include: interferon response factor 1 (*irf1*), several MHC, effectors of the cytotoxic T-cell response including perforin (*prf1*), complement components (*c3*, *c1qa*) and cytokines (*il12*).

To discover programs of responses to invasive melanoma at its initial stage, we tested for significant participation among signaling and metabolic pathways, molecular networks, and biological processes that are most perturbed in these juvenile melanoma fish [22]. A total of 46 pathways were considered further (p-value <0.01; Table D in S1 File). One of the significantly scored pathways was melanocyte development and pigmentation (Fig 2; Table D in S1 File), again validating our intact-fish approach for understanding organism-wide changes to gene expression that are likely to be highly relevant for indirect correlation to human melanoma progression. Two of the other highest scoring pathways were antigen presentation (p-value 0.0000014) and cytotoxic T lymphocyte-mediated apoptosis (p-value 0.000251). Antigen presentation is a vital process of the immune system with many cellular participants acting as sentinels that oscillate between detecting self versus entities that disrupt body homeostasis, i.e., cancer. We found members *b2m*, *cd74*, *hla-dqa1*, *hla-drb1*, *psmb9*, and *tap2* of this pathway to all be down-regulated in melanoma fish (Table A in S1 File). After manual curation, a total of 6 of the top 10 scoring pathways (p-value <0.001; Table D in S1 File) were affiliated with the immune system. To our knowledge this phenomenon was not observed in previous studies of the fish melanoma models where dissected tumors were analyzed [11, 34, 35].

To expand our systemic knowledge of whole body melanoma transcriptomes, we sought to elucidate the putative upstream regulators of melanoma-expressed genes for probable states of activation or inhibition that may provide testable hypotheses of downstream effects on melanoma sustainability (Table 1). We used the inferred activation states of transcriptional regulators given the observed differential state of genes in our medaka melanoma model for this purpose [22]. A z-score statistic was used to predict expression states, activated or inhibited, that we set at -2 or +2 to reach significance. Given this threshold, we found 17 and 16 molecules that predict an inhibitory or activated effect of a given regulator, respectively, on various medaka genes expressed in this whole body melanoma model (Table 1). A cumulative list of all molecules that met this statistical criteria are provided for further review (see S2 File). The three molecules of highest inferred inhibitory significance were *ifnγ*, *cd40lg*, and *tnf-alpha*. Interestingly, we found that *ifnγ* is the top ranked upstream regulator of genes expressed in medaka developing melanoma (z-score -5.08). These putatively *ifnγ* regulated genes (33 total) span several overlapping molecular pathways as shown in a network based presentation (Fig A in S1 File). The majority (82%) of the 33 differentially expressed medaka genes in melanoma fish were down-regulated as part of our curated *ifnγ* regulatory network and all but one, *ehf*, agree with expectations based on the Ingenuity curated expression database.

**Table 1:** Estimated activation state for inferred melanoma gene network regulators.

Upstream Regulator	Molecule Type	Predicted Activation State	Activation z-score	Medaka melanoma gene network count
IFN-gamma	cytokine	Inhibited	-5.082	*33*
TNF	cytokine	Inhibited	-3.173	24
CD40LG	cytokine	Inhibited	-2.962	12
LPS	chemical drug	Inhibited	-2.774	24
IRF7	transcription regulator	Inhibited	-2.63	7
IL1B	cytokine	Inhibited	-2.626	17
IL15	cytokine	Inhibited	-2.583	11
STAT1	transcription regulator	Inhibited	-2.476	12
LFNAR	group	Inhibited	-2.449	6
IRF1	transcription regulator	Inhibited	-2.361	9
CD40	transmembrane receptor	Inhibited	-2.356	6
IL2	cytokine	Inhibited	-2.253	12
Aldesleukin	biologic drug	Inhibited	-2.219	5
RELA	transcription regulator	Inhibited	-2.13	15
IFN-alpha	group	Inhibited	-2.095	8
Cisplatin	chemical drug	Inhibited	-2.064	10
Inosine	chemical	Inhibited	-2	4
Alefacept	biologic drug	Activated	2	4
OTX2	transcription regulator	Activated	2	4
CDKN2A	transcription regulator	Activated	2	5
SB203580	chemical—kinase inhibitor	Activated	2.144	6
LNS1	other	Activated	2.172	5
PRDM1	transcription regulator	Activated	2.183	5
Mibolerone	chemical drug	Activated	2.2	5
PPARG	ligand-dependent nuclear receptor	Activated	2.213	5
MAPK1	kinase	Activated	2.219	6
APOE	transporter	Activated	2.226	7
Forskolin	chemical toxicant	Activated	2.236	15
Akt	group	Activated	2.236	5
RICTOR	other	Activated	2.236	5
CD3	complex	Activated	2.447	15
PPARA	ligand-dependent nuclear receptor	Activated	2.651	12
IL10	cytokine	Activated	2.751	9

**Fig 3:**
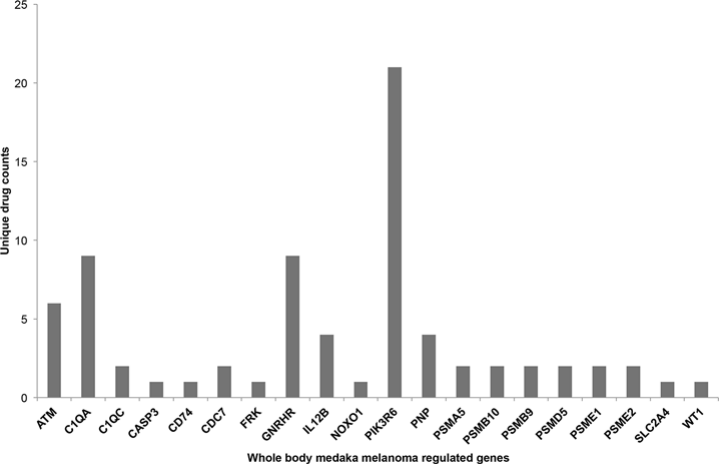
Number of unique drugs predicted to target each known medaka melanoma differentially expressed gene. The drug list is presented in full in S3 File.

Thus far, fish have provided useful models for furthering our understanding of melanoma biology and basic molecular mechanisms and for screening of potential drugs that act directly on the tumor [36]. When all differentially regulated expression candidate genes with matching human gene orthologs (n = 233) were examined for known drug to gene interactions, we found 10 genes with 73 unique drug interactions [23], with the phosphatidylinositol kinase gene *pik3r6* contributing 21 unique drug interactions (Fig 3). In addition, *pik3r6* displays recurrent somatic mutations in human melanoma and would thus be considered a high priority target in future experiments designed to utilize drugs that target this gene in our melanoma fish and observe therapeutic outcomes [29].

## Discussion

The effects of genetic background on human malignancy progression is an important but poorly understood phenomenon [37]. Our system provides a significant opportunity to explore this problem and already its investigation has led to the identification of several melanoma genes, including osteopontin [38], which now serves as a clinical marker for human disease progression [39]. Among fish models, medaka presents as a unique feature the availability of many highly inbred strains that allow one to study cancer formation in animals of the same or a variety of different genetic backgrounds. Furthermore, the *xmrk* transgene on different genetic backgrounds provides different types of melanoma [17] and transforms mouse and human melanocytes [26, 27] that result in highly similar biochemical and genetic changes [10, 14], making this system ideal for identifying both genetic modifiers of tumor type and drugs tailored to different genotypes. The pigment lesions in our mitf:xmrk transgenic medaka at the time of RNA-seq sampling are clearly not just “local hyperpigmentation” in the dermis but an aggressive malignancy that is located predominantly at extracutaneous sites, mainly as invasive growth between the muscle fibers and bones. Melanoma cells contain black pigment, and due to the transparency of the juvenile medaka, the area covered by melanin inside the fish in the trunk is a good proxy for estimating the size of the tumor. Moreover, the single homologous gene driver and the short time window in which tumors fully invade the whole body excludes many types of secondary (passenger) mutations that might occur. To exploit this situation, we explored the use of whole body transcriptome profiles to catalog gene candidates for therapeutic intervention and to discover the body’s canonical pathway responses to highly invasive melanoma.

The whole-body transcriptomes established in our study from melanoma-bearing transgenic medaka readily detected changes in canonical pathways known from studies on human melanoma [40], but also show a clear differentiation from earlier RNA-seq analysis of melanoma biopsies from adult medaka fish [10]. We identified a considerable number of genes that were not detected earlier when analyzing tumors in isolation [10]. For instance, *snord14c* showed the strongest down-regulated state in the whole body melanoma transcriptome, yet its role in melanoma formation is not understood to our knowledge, and thus identifies a new putative candidate for the further understanding of the molecular mechanisms of melanoma progression, perhaps highlighting a role for RNA methylation. The *fkbp5* gene is another example of a potential contributor to melanoma dissemination in this medaka expansion model. Recent studies highlighted *fkbp5* in the transformation of transforming growth factor-β from a tumor suppressor to pro-metastatic aggressor, facilitating cancer cells ability to spread to distant sites from the original primary tumor site [31].

Our whole body set of differentially expressed genes, critically collected when melanoma is in invasive mode, allowed us to search for molecular pathways that are significantly enriched in the melanoma developing fish. Given the transgenic initiation of melanocyte malignancy in our model, it was predicted at least some known genes associated with this pathway would be differentially regulated. The high scoring melanocyte pathway clearly shows that our whole body transcriptome measures were sufficient to detect modulation of some member genes, such as *tyrp1*, over whole body signal noise. The increase of *tyrp1* expression is interesting since melanin synthesis, melanogenesis and melanin pigment affect the behavior of normal and malignant melanocytes. In melanoma cells melanogenesis is related to shorter survival of patients [41]. More importantly, our results shine new light on the direct relevance of the mechanisms by which immune gene networks respond or fail to respond to the expanding population of malignant melanocytes and are likely highly relevant to human melanoma pathogenesis. As a consequence of melanocyte proliferation and spread, we hypothesize that intact body systems attempt to identify and kill malignant melanocytes, but too often this alarm to the immune system is insufficient to overcome a fast growth tumor trajectory, especially if cellular cytoskeleton dynamics are not optimum. A heightened immune response could be compromised by reported immunosuppressive properties of malignant melanocytes [42]. In fact, an important immune pathway, antigen presentation, was found to be enriched for several differentially expressed medaka member genes and we observed down-regulation for all in the melanoma state. This may indicate mechanisms of immune escape of the fish pigment cell tumor in its initial stage and resembles conditions that have been noted also in human melanoma [43]. Further expansion of our list of statistically significant genes regulated within the known immune signaling pathways is likely with deeper sequencing per whole body melanoma isolate.

The inability of the immune system to detect and react with sufficient intensity to check progressive melanoma has confounded scientists. Some evidence suggests that melanoma has an immunosuppressive capability [42, 44]. In particular, intermediates in melanin synthesis,e.g. L-DOPA have immunosuppressive capability [45, 46]. We found several genes in our differentially expressed gene set that, when examined at the network level, broadly suggest immune system suppression; it will be important, however, to differentiate the mechanisms of immunosuppressive or atypical inflammatory responses for these candidates. Within several immune pathway classifications, such as antigen presentation, dendritic cell maturation, and cytotoxic T lymphocyte-mediated apoptosis, we observe all but one gene showing decreased expression in medaka melanoma. Together with the finding that most genes involved in immune system pathways were down-regulated in this early progression phase of fish melanomas, this result suggests that immune evasion might be important for the invasive success of malignant melanoma. Interestingly, a soluble form of *pmel* can protect melanoma cells from antibody-mediated immunity [47], while the membrane-bound form activates the immune response via dendritic cells [48]. While the transcript isoform of this gene was highly up-regulated in our melanoma fishes, protein form responses will need to be measured to fully understand the immunomodulatory consequences. Of note, in this study we believe the immune suppressive state that we observed at a very early stage (3–4 weeks) of tumor formation excludes the explanation that immune evasion is due to additional acquired mutations; rather, it is an indigenous feature of melanoma cells.

Utilizing prior knowledge of expected directional relationships between transcriptional regulators of all types (drugs, transcription factors, small molecules, etc.) and downstream expressed genes is a powerful model for high-level melanoma gene network inference (Fig A in S1 File). Our analysis of known upstream regulators strongly suggests that melanoma fish experience a robust suppression of the *ifnγ* responsive gene network, the highest scoring network in this study. Recently, an unexpected connection between the immune cytokine *ifnγ* and skin pigmentation homeostasis was found [49] mediated through the transcription factor *irf1*, a gene that we find consistently downregulated in the melanoma developing medaka. High-dose interferon has been used for nearly two decades as adjuvant treatment of patients with melanoma who have undergone a complete surgical resection but who are considered to be at a high risk of relapse. Evidence leading to interferon therapy was based on significantly improved relapse-free survival and marginally improved overall survival. Subsequent large, randomized trials have not been able to reproduce a benefit in overall survival, yet interferon treatment continues to be a first option for those patients [50]. *Stat5*, which was first identified as a melanoma-relevant gene in our fish melanoma model [51], is now used in the clinic to elucidate mechanisms of resistance to interferon treatment in human patients [25]. Collectively, our network inferred immune-system gene candidates could provide points of entry for therapeutics to elevate the immune response of the host to progressing melanoma or conversely, to subdue putative immunosuppression by malignant melanocytes. Such molecular insight will be challenging without our whole-body approach.

Importantly, our whole-body expression screen identified candidates for future drug screens, in particular immunoregulatory molecules. Drug screen outcomes will require network or systems biology interpretation because some genes participate in multiple pathways, for example, phosphatidylinositol kinases act in leukocyte extravasation and antigen pattern recognition pathways. A total of 11 aberrant medaka melanoma gene expression candidates found in our study, some examples include *aadac*, *dhdh*, *dusp13*, and *pik3r6*, have experienced missense (amino acid substitution) mutations in human melanoma genomes [52]. Despite the unknown clinical relevance of these mostly missense mutations in melanoma patients, we suggest further investigation of any drugs known to target these proteins translated from differentially expressed genes in our fish melanoma model, but not currently used to treat human melanoma, is needed to test transcript outcome in isolation and synergy, i.e., drug combinations [29]. We contend that a reduced transcript network, that indirectly mimics the transcriptional disease signature, could be used to cost effectively screen drugs in a high throughput system.

In summary, building genetic networks that adequately represent whole body transcriptome responses to invasive cancer is crucial to the exploration of secondary pathways for melanoma suppression. Cancer genome-sequencing studies have identified recurring mutational signatures in various cancers including melanoma, but the corresponding immunological signatures of tumors are highly variable and are far from well understood. Closing this knowledge gap is likely to be important, given that infiltration of immune cells into tumors is correlated with positive prognoses [53, 54]. A better understanding of the complex interactions between cancer cells and the immune system is likely to lead to improvements in current therapeutic approaches and to spur the development of novel therapies. Our study 1) illustrates how focusing only on the tumor itself might leave unknowns that are revealed when whole animals are taken into account; 2) shows how the tumor can affect non-tumor body systems; and 3) provides insights into new potential therapeutic targets. The whole body transcriptome based characterization of the medaka system can serve as model for further research on melanoma tumor immunology.

## Supporting Information

S1 File (DOCX)

S2 File (XLS)

S3 File (XLS)
