# Supplementary material for: Whole Body Melanoma Transcriptome Response in Medaka
Source: PLoS One. 2015 Dec 29;10(12):e0143057. doi: 10.1371/journal.pone.0143057 (PMC4699850; doi:10.1371/journal.pone.0143057)
Supplement: S1 File — (DOCX) [file pone.0143057.s001.docx]

**Table A**. Differentially expressed medaka melanoma genes with human orthologs used for canonical pathway enrichment analyses.

| **Gene Symbol** | **Log ratio expression** | **Adjusted p-value** |
| --- | --- | --- |
| FKBP5 | 2.486000068 | 1.79E-008 |
| SLC16A12 | 2.242127333 | 2.45E-007 |
| ASB14 | 2.200448132 | 1.10E-010 |
| GSTT2 | 1.983005389 | 2.64E-005 |
| OPN4 | 1.965388296 | 9.20E-006 |
| TAS1R1 | 1.922026746 | 3.00E-006 |
| ZBTB16 | 1.830668744 | 0.000774727 |
| STC1 | 1.789649551 | 4.60E-005 |
| EPDL | 1.752016421 | 0.000378029 |
| PMEL | 1.645275016 | 2.31E-005 |
| SLC13A3 | 1.631442745 | 4.30E-008 |
| FRRS1 | 1.609995115 | 3.34E-007 |
| WDR64 | 1.584079466 | 0.000200442 |
| ELOVL5 | 1.546156358 | 0.001322876 |
| DDX17 | 1.53980835 | 7.83E-011 |
| CREB3L4 | 1.533336044 | 4.86E-007 |
| MIR206 | 1.532393988 | 0.000977263 |
| TYRP1 | 1.526479352 | 0.000286539 |
| MIR133B | 1.516310881 | 0.000258015 |
| SLC24A5 | 1.514611595 | 0.000370211 |
| OTOS | 1.50865703 | 7.49E-006 |
| PNP | 1.476967987 | 3.57E-005 |
| ZNHIT1 | 1.438936728 | 0.000215591 |
| TMEM8C | 1.402321435 | 0.008969102 |
| FADS2 | 1.374965854 | 0.010669895 |
| GATM | 1.352760796 | 0.003153433 |
| LAMB3 | 1.291929397 | 0.013946701 |
| ABCC12 | 1.253329661 | 0.006471452 |
| HEPACAM | 1.24667904 | 4.47E-005 |
| JAM2 | 1.245557454 | 0.000491513 |
| NAT1 | 1.236540896 | 0.002407462 |
| PER1 | 1.228894948 | 3.95E-008 |
| NEK10 | 1.207783207 | 0.004370872 |
| TMEM174 | 1.207056691 | 0.000796227 |
| TRIM2 | 1.206381842 | 0.007415009 |
| MIR103A2 | 1.206350838 | 0.010701564 |
| PANK1 | 1.196497209 | 0.002932282 |
| RAI1 | 1.15865008 | 0.01149224 |
| NGS | 1.158398823 | 0.002103784 |
| SLC25A35 | 1.153923653 | 0.007715943 |
| IKBIP | 1.139933947 | 0.002505981 |
| HSD11B2 | 1.13898461 | 0.001311873 |
| OCA2 | 1.135957567 | 0.000144971 |
| GAMT | 1.130895194 | 0.004776778 |
| CIART | 1.130683438 | 0.00032108 |
| PAQR8 | 1.127431971 | 0.00551005 |
| PEAR1 | 1.125331493 | 0.012650014 |
| ATM | 1.120637271 | 0.001470399 |
| SLC22A6 | 1.118941623 | 0.017145981 |
| SLC34A2 | 1.102532544 | 0.000136703 |
| AP000350.10 | 1.097993239 | 0.005051264 |
| STK35 | 1.09727347 | 0.012335576 |
| FRAS1 | 1.095873466 | 6.35E-005 |
| DSC1 | 1.092274986 | 0.00551005 |
| PIPOX | 1.084457781 | 0.000459678 |
| SLC2A4 | 1.074712804 | 0.005051264 |
| AADAC | 1.070214192 | 0.005542456 |
| SLC47A1 | 1.066890365 | 0.000502698 |
| SCEL | 1.06325414 | 0.004776778 |
| EDNRB | 1.063003374 | 0.000491513 |
| WT1 | 1.057701431 | 0.014857421 |
| LCAT | 1.056050006 | 0.013238754 |
| NCALB | 1.049653548 | 0.001297723 |
| PCOLCE2 | 1.037912623 | 0.009306576 |
| ARMC2 | 1.031025891 | 0.001831857 |
| SYNGR1 | 1.007821533 | 0.012938002 |
| HIF3A | 1.0042269 | 0.004793441 |
| SLC7A8 | 1.003898516 | 0.00258738 |
| TSPAN5 | 1.001099347 | 0.011025426 |
| KLF11 | 0.999147806 | 0.008697923 |
| LOXL2 | 0.991682779 | 0.007998692 |
| GPR61 | 0.987064247 | 0.014087284 |
| COL11A1 | 0.979241581 | 0.000912707 |
| NAGS | 0.965792315 | 0.017784883 |
| ABCA1 | 0.954357415 | 0.008596525 |
| SNAI1 | 0.952924152 | 0.0025385 |
| CRABP2 | 0.945484069 | 0.005662228 |
| AOC1 | 0.943957817 | 0.000857399 |
| SOX11 | 0.908149631 | 0.005917496 |
| ULK2 | 0.899908516 | 0.001172332 |
| MFI2 | 0.898246081 | 0.016952103 |
| DSEL | 0.895567851 | 0.013620705 |
| FAHD1 | 0.894454062 | 0.011500339 |
| EDNR | 0.894099675 | 0.0156365 |
| GRK1 | 0.891756224 | 0.012624054 |
| RAB34 | 0.89014695 | 0.004293413 |
| MIEF1 | 0.869814662 | 0.005790082 |
| EFEMP2 | 0.859537527 | 0.003947779 |
| SYDE1 | 0.850186658 | 0.001791234 |
| EHD3 | 0.848768414 | 0.001899492 |
| TMTC2 | 0.840611817 | 0.013620705 |
| SRSF2 | 0.814445158 | 0.017977792 |
| HIATL2 | 0.79103496 | 0.011311069 |
| UGT2A1 | 0.769165353 | 0.007715943 |
| RBM24 | 0.716691051 | 0.014209093 |
| MBD1 | -0.734706366 | 0.016643766 |
| PSME1 | -0.74342116 | 0.010882545 |
| EIF5B | -0.751313325 | 0.012162779 |
| COG4 | -0.774652547 | 0.010342277 |
| PSMD5 | -0.77934128 | 0.012686018 |
| TMPRSS4 | -0.77998521 | 0.007235594 |
| CALCOCO2 | -0.819811839 | 0.011453794 |
| FILIP1 | -0.823105683 | 0.003421083 |
| PTPN6 | -0.840461497 | 0.002103784 |
| CHD1L | -0.853468076 | 0.015486426 |
| STAT4 | -0.859506499 | 0.007818618 |
| SEMA4G | -0.862033555 | 0.019978284 |
| PLCG2 | -0.866119694 | 0.011500339 |
| PTPN7 | -0.87415261 | 0.000177261 |
| PIBF1 | -0.877870267 | 0.01554764 |
| RIOK2 | -0.883386525 | 0.011068469 |
| ACTR6 | -0.888534669 | 0.009306576 |
| MARVELD2 | -0.909087633 | 0.008940263 |
| XPA | -0.913363472 | 0.013580516 |
| AVIL | -0.914795966 | 0.013620705 |
| CD74 | -0.917671912 | 0.003986827 |
| CNGA1 | -0.918528509 | 0.008611646 |
| PADI2 | -0.930872271 | 0.006383074 |
| CA4 | -0.933103163 | 0.010214552 |
| PLAC8 | -0.933982474 | 0.00937704 |
| GALE | -0.95006014 | 0.010639904 |
| C1QA | -0.952594488 | 0.016663749 |
| GCNT1 | -0.954553616 | 0.000265781 |
| HCLS1 | -0.972813168 | 0.003893904 |
| HIP1R | -0.986820375 | 0.011452852 |
| CUL5 | -0.98786665 | 0.004758255 |
| TUBGCP4 | -0.988650151 | 0.004771175 |
| CLSPN | -0.997159331 | 0.015292716 |
| GLULC | -1.011169399 | 0.006376302 |
| ADAP2 | -1.016150214 | 0.003893904 |
| CDC7 | -1.032301725 | 0.013610397 |
| FADD | -1.032821219 | 0.001297723 |
| PBXIP1B | -1.037132427 | 0.007998692 |
| DHDH | -1.050726928 | 0.001622254 |
| PSME2 | -1.055414697 | 0.002954257 |
| GIMAP5 | -1.067732253 | 0.013070982 |
| IFI30 | -1.076388722 | 0.000317732 |
| PARGL | -1.08814682 | 0.000123035 |
| ERAP2 | -1.105458434 | 0.000287548 |
| KIAA1598 | -1.110062242 | 0.016306568 |
| CHIA | -1.111455947 | 0.001251515 |
| DLG4 | -1.112110459 | 0.013014077 |
| C4B | -1.125156215 | 0.011144288 |
| MFAP4 | -1.126516587 | 0.018378261 |
| TRIM29 | -1.13210389 | 0.004384129 |
| TRIM35 | -1.138423991 | 0.012938002 |
| PSMA5 | -1.146509623 | 7.62E-005 |
| LCN15 | -1.151873099 | 0.001159044 |
| SUZ12 | -1.154382703 | 0.001021281 |
| IL12B | -1.159222759 | 0.008969102 |
| HLA-DRB1 | -1.159590049 | 0.013620705 |
| PSMB9 | -1.168288834 | 0.00302696 |
| PSMB10 | -1.170138547 | 0.00131539 |
| MCTP2 | -1.176016396 | 0.000236936 |
| C1QC | -1.181325356 | 0.004758778 |
| TWF2 | -1.18878695 | 0.003515746 |
| GBGT1 | -1.192186481 | 2.31E-005 |
| MFAP2 | -1.194864155 | 0.009656738 |
| MYH8 | -1.196702892 | 0.013779957 |
| NME4 | -1.198180701 | 0.000317732 |
| MYH6 | -1.198456497 | 0.001159044 |
| PIK3R6 | -1.202266724 | 0.004144191 |
| DUSP13 | -1.216376149 | 0.003001661 |
| TC2N | -1.220836338 | 0.000190582 |
| S100A3 | -1.22740878 | 0.00041026 |
| HMBS | -1.237182419 | 0.008545655 |
| C3 | -1.241257286 | 0.011068469 |
| B2M | -1.243587649 | 4.87E-007 |
| LMNL3 | -1.262608775 | 0.004599286 |
| C19orf52 | -1.26454919 | 0.012143196 |
| DPEP1 | -1.26609737 | 0.007945054 |
| GRK5 | -1.281007189 | 0.013295108 |
| OLFM4 | -1.285564131 | 0.001423479 |
| DNAJA4 | -1.286823681 | 0.002868389 |
| GPALPP1 | -1.29159643 | 0.002277807 |
| GNRHR | -1.292331592 | 0.008062199 |
| SCO1 | -1.307325687 | 0.00032108 |
| ENDOD1 | -1.317693059 | 0.000119205 |
| NEURL3 | -1.326459495 | 0.001297723 |
| FAM53C | -1.330285363 | 0.014882256 |
| ANKRD34B | -1.331382023 | 0.007945054 |
| ETV7 | -1.33663484 | 4.00E-007 |
| UDP | -1.3424663 | 0.009691006 |
| CYBA | -1.353187076 | 3.97E-009 |
| PROKR2 | -1.363746245 | 0.008978978 |
| RNH1 | -1.365055662 | 0.001640951 |
| NFIL3 | -1.370547265 | 0.004624475 |
| ZG16B | -1.375555458 | 0.000123035 |
| KRT78 | -1.395536607 | 0.016643766 |
| SSR3 | -1.428645427 | 0.002071346 |
| TRPV1 | -1.428725795 | 0.000518201 |
| CRYBB3 | -1.439094122 | 0.000258015 |
| CRYBB2 | -1.456252982 | 0.000912391 |
| SNORD88B | -1.490780468 | 0.013580516 |
| DNASE1L2 | -1.497529473 | 0.000177261 |
| GBA3 | -1.508871293 | 0.006106322 |
| ZNF683 | -1.547254513 | 0.00032108 |
| PTK6 | -1.564427231 | 2.37E-007 |
| ALOX15B | -1.567488888 | 7.93E-005 |
| BDKRB2 | -1.56761595 | 0.000361779 |
| DDIT4L | -1.5763649 | 0.000258015 |
| TNFRSF14 | -1.577970982 | 1.02E-005 |
| RPZ4 | -1.584318093 | 8.14E-009 |
| DTX3L | -1.589925496 | 0.00303032 |
| TECTA | -1.613818117 | 0.001558216 |
| CCDC171 | -1.659968424 | 4.03E-005 |
| LPO | -1.687319965 | 0.002851348 |
| IRF10 | -1.697309055 | 1.13E-006 |
| EHF | -1.733362822 | 2.98E-007 |
| GCNT3 | -1.754415474 | 0.000209755 |
| SLC28A3 | -1.766774757 | 0.00017615 |
| TAP2 | -1.771266046 | 1.10E-005 |
| PARP15 | -1.792010438 | 7.39E-006 |
| TAPBPL | -1.795795052 | 2.22E-006 |
| IRF1 | -1.795966567 | 0.000213812 |
| METTL21C | -1.829792139 | 5.63E-005 |
| METTL10 | -1.850168289 | 6.35E-006 |
| APCS | -1.86359725 | 1.08E-006 |
| NOX1 | -1.879122598 | 1.58E-010 |
| CASP3 | -1.936051273 | 2.99E-005 |
| FRK | -1.969643809 | 2.01E-012 |
| CTSG | -2.008814917 | 7.39E-006 |
| APOD | -2.016375667 | 6.08E-005 |
| KLHL33 | -2.03061979 | 9.85E-007 |
| PTPRH | -2.03142875 | 4.53E-009 |
| B3GALTL | -2.144482339 | 4.92E-008 |
| FBL | -2.296372183 | 5.18E-016 |
| PLEKHF1 | -3.131053029 | 1.26E-022 |
| NOXO1 | -3.268455654 | 4.90E-015 |
| PRF1 | -3.998055995 | 2.53E-023 |
| VCANA | -5.380485413 | 2.04E-046 |
| HLA-DQA1 | -5.472976625 | 4.80E-054 |
| SNORD14C | -5.671318975 | 2.25E-048 |

**Table B**. Validation of differential gene expression by qPCR on 10 healthy and 10 melanoma fishes

| Gene | P-value qPCR | qPCR Fold Change | P-value RNAseq | Log2 Fold change |
| --- | --- | --- | --- | --- |
| IRF1 | 0.00027 | 0.18 | 1.41 E-06 | -1.79 |
| STAT4 | 0.682 | 1.09 | 0.00011 | -0.86 |
| ATM | 0.041 | 1.87 | 1.54 E-05 | 1.12 |
| STC1L | 0.053 | 1.77 | 2.17 E-07 | 1.79 |
| SLC24A5 | 0.00027 | 4.87 | 2.88 E-06 | 1.51 |
| EPDL1 | 0.000411 | 7.35 | 2.97 E-06 | 1.75 |
| TLR2 | 0.00019 | 0.004 | 0.0088 | -0.60 |
| GADD45A | 0.9674 | 0.90 | 0.0027 | 1.20 |

**Table C**. Differentially expressed medaka melanoma genes with human orthologs that display recurrent somatic mutations in human melanoma.

Medaka ID Log ratio Human ID Gene name Mutation type

ENSORLG00000005954 1.07 ENSG00000114771 AADACL2 missense

ENSORLG00000014988 1.25 ENSG00000140798 ABCC11 missense

ENSORLG00000010192 -1.02 ENSG00000184060 ADAP2 missense

ENSORLG00000017255 -1.33 ENSG00000089847 ANKRD24 missense

ENSORLG00000014827 -1.45 ENSG00000244752 CRYBB2 missense

ENSORLG00000013210 -1.05 ENSG00000104808 DHDH missense

ENSORLG00000017106 1.29 ENSG00000134765 DSC1 splice_site

ENSORLG00000009502 -1.22 ENSG00000079393 DUSP13 missense

ENSORLG00000008714 -1.10 ENSG00000164308 ERAP2 missense

ENSORLG00000014928 -1.17 ENSG00000140563 MCTP2 missense

ENSORLG00000003102 -1.21 ENSG00000174083 PIK3R6 missense

ENSORLG00000012315 -1.79 ENSG00000139192 TAPBPL missense

ENSORLG00000004326 1.52 ENSG00000107165 TYRP1 missense

ENSORLG00000014987 0.90 ENSG00000083290 ULK2 missense

|  |  |  |  |  |
| --- | --- | --- | --- | --- |
|  |  |  |  |  |

**Table D**. Canonical pathway enrichment analysis of expressed medaka melanoma genes

| Ingenuity Canonical Pathways | Regulated genes | P-value |
| --- | --- | --- |
| Antigen Presentation Pathway | B2M,PSMB9,HLA-DRB1,HLA-DQA1,CD74,TAP2 | 1.41254E-06 |
| Dendritic Cell Maturation | STAT4,B2M,HLA-DRB1,IL12B,PLCG2,PIK3R6,HLA-DQA1,CREB3L4,ATM | 6.60693E-05 |
| Type I Diabetes Mellitus Signaling | FADD,PRF1,HLA-DRB1,CASP3,IL12B,HLA-DQA1,IRF1 | 0.000107152 |
| Melanocyte Development and Pigmentation Signaling | TYRP1,PTPN6,PLCG2,PIK3R6,CREB3L4,ATM | 0.000190546 |
| Cytotoxic T Lymphocyte-mediated Apoptosis of Target Cells | B2M,FADD,PRF1,CASP3 | 0.000251189 |
| Retinoic acid Mediated Apoptosis Signaling | FADD,PARP15,CASP3,CRABP2,IRF1 | 0.000489779 |
| Growth Hormone Signaling | PTPN6,PLCG2,PIK3R6,SLC2A4,ATM | 0.000724436 |
| IL-4 Signaling | PTPN6,HLA-DRB1,PIK3R6,HLA-DQA1,ATM | 0.000977237 |
| Role of Pattern Recognition Receptors in Recognition of Bacteria and Viruses | IL12B,PLCG2,PIK3R6,C1QC,C1QA,ATM | 0.001513561 |
| Tumoricidal Function of Hepatic Natural Killer Cells | FADD,PRF1,CASP3 | 0.001584893 |
| UVA-Induced MAPK Signaling | PARP15,CASP3,PLCG2,PIK3R6,ATM | 0.001778279 |
| IL-15 Production | PTK6,FRK,IRF1 | 0.002238721 |
| Production of Nitric Oxide and Reactive Oxygen Species in Macrophages | PTPN6,PLCG2,CYBA,PIK3R6,IRF1,ATM,APOD | 0.002344229 |
| IL-12 Signaling and Production in Macrophages | STAT4,IL12B,PIK3R6,IRF1,ATM,APOD | 0.002398833 |
| Myc Mediated Apoptosis Signaling | FADD,CASP3,PIK3R6,ATM | 0.002454709 |
| ILK Signaling | MYH6,CASP3,MYH8,SNAI1,PIK3R6,CREB3L4,ATM | 0.002454709 |
| Glycine Degradation (Creatine Biosynthesis) | GAMT,GATM | 0.003235937 |
| Protein Ubiquitination Pathway | B2M,PSMB9,PSME1,PSMB10,PSMA5,PSMD5,PSME2,TAP2 | 0.003890451 |
| eNOS Signaling | BDKRB2,CASP3,PLCG2,PIK3R6,CNGA1,ATM | 0.004168694 |
| Macropinocytosis Signaling | PLCG2,PIK3R6,RAB34,ATM | 0.004365158 |
| Erythropoietin Signaling | PTPN6,PLCG2,PIK3R6,ATM | 0.004786301 |
| Tec Kinase Signaling | STAT4,FADD,PLCG2,PIK3R6,FRK,ATM | 0.005011872 |
| T Helper Cell Differentiation | STAT4,HLA-DRB1,IL12B,HLA-DQA1 | 0.005370318 |
| JAK/Stat Signaling | STAT4,PTPN6,PIK3R6,ATM | 0.005370318 |
| Renin-Angiotensin Signaling | PTPN6,PLCG2,PIK3R6,NOX1,ATM | 0.005888437 |
| FLT3 Signaling in Hematopoietic Progenitor Cells | STAT4,PIK3R6,CREB3L4,ATM | 0.005888437 |
| Complement System | C4A/C4B,C1QC,C1QA | 0.005888437 |
| Prolactin Signaling | PLCG2,PIK3R6,IRF1,ATM | 0.006456542 |
| PKCθ Signaling in T Lymphocytes | HLA-DRB1,PLCG2,PIK3R6,HLA-DQA1,ATM | 0.00676083 |
| Glycine Cleavage Complex | OCA2,NAD+ | 0.00676083 |
| Role of PKR in Interferon Induction and Antiviral Response | FADD,CASP3,IRF1 | 0.00691831 |
| CD28 Signaling in T Helper Cells | PTPN6,HLA-DRB1,PIK3R6,HLA-DQA1,ATM | 0.007079458 |
| Huntington's Disease Signaling | PSME1,CASP3,PIK3R6,PSME2,DLG4,CREB3L4,ATM | 0.008317638 |
| FcγRIIB Signaling in B Lymphocytes | PLCG2,PIK3R6,ATM | 0.009549926 |
| Docosahexaenoic Acid (DHA) Signaling | CASP3,PIK3R6,ATM | 0.009549926 |
| Endothelin-1 Signaling | CASP3,LCAT,EDNRB,PLCG2,PIK3R6,ATM | 0.01 |
| Granzyme B Signaling | PRF1,CASP3 | 0.01023293 |
| Allograft Rejection Signaling | B2M,PRF1,HLA-DRB1,HLA-DQA1 | 0.01023293 |
| Sperm Motility | LCAT,PLCG2,PTK6,CNGA1,FRK | 0.010715193 |
| MSP-RON Signaling Pathway | IL12B,PIK3R6,ATM | 0.010715193 |
| CTLA4 Signaling in Cytotoxic T Lymphocytes | B2M,PTPN6,PIK3R6,ATM | 0.010715193 |
| FGF Signaling | PTPN6,PIK3R6,CREB3L4,ATM | 0.011220185 |
| Graft-versus-Host Disease Signaling | PRF1,HLA-DRB1,HLA-DQA1 | 0.012022644 |
| Autoimmune Thyroid Disease Signaling | PRF1,HLA-DRB1,HLA-DQA1 | 0.013489629 |
| Virus Entry via Endocytic Pathways | B2M,PLCG2,PIK3R6,ATM | 0.013803843 |
| Leukocyte Extravasation Signaling | PLCG2,CYBA,PIK3R6,JAM2,NOX1,ATM | 0.014791084 |

**Fig A**. Genes known to be linked to IFN regulatory networks. All gene interactions were derived from an extensive literature curated database available at Ingenuity. Red or green colored gene symbols show increased or decreased expression, respectively, in melanoma fishes compared to wild types. Blue dashed lines indicate predicted inhibition and orange activation, respectively . Yellow dashed lines indicate the observed expression directionality was not expected when comparing to the known action in previous studies.
